# Radiation dosimetry of florbetapir F 18

**DOI:** 10.1186/2191-219X-4-4

**Published:** 2014-01-08

**Authors:** Abhinay D Joshi, Michael J Pontecorvo, Lee Adler, Michael G Stabin, Daniel M Skovronsky, Alan P Carpenter, Mark A Mintun

**Affiliations:** 1 Avid Radiopharmaceuticals, Inc., 3711 Market Street, 7th Floor, Philadelphia, PA 19104, USA; 2 Adler Institute for Advanced Imaging, Jenkintown, PA 19046, USA; 3 Department of Radiology and Radiological Sciences, Vanderbilt University, Nashville, TN 37235, USA; 4 Department of Radiology, University of Pennsylvania, Philadelphia, PA 19104, USA

## Abstract

**Background:**

Florbetapir is one of several ^18^F-labeled amyloid plaque imaging tracers for positron emission tomography (PET). As the bio-distribution and radiation dose of PET tracers in human research are important for estimating the relative risks and benefits, a study was conducted to obtain this information on florbetapir.

**Methods:**

Nine cognitively normal subjects (six females and three males, age 58 ± 10 years, weight 81 ± 17 kg) received an intravenous bolus injection of 395 ± 27.9 MBq of florbetapir, and whole-body emission scans were performed over approximately 6 h. Computed tomography scans were acquired for attenuation correction. Volumes of interest (VOIs) for source organs including the brain, liver, lung, heart wall, and vertebrae were defined on the PET images. The VOIs of the gallbladder, urinary bladder, and large and small intestines were also defined. Using reference man organ volumes (ICRP 30), total activity was calculated per organ for each time point. The resultant time-activity curves (TACs) were fitted with constrained exponentials. Kinetic data were entered into OLINDA/EXM software to calculate dose estimates; the dynamic urinary bladder and ICRP 30 GI tract models were employed. The effective dose (ED) for each subject was estimated from the acquired data using the adult model.

**Results:**

The mean ED determined for nine healthy volunteers was 18.60 ± 4.26 μSv/MBq or 6.88 mSv for a 370-MBq dose. The organs that received the highest radiation absorbed doses were the gallbladder, upper large intestine, small intestine, liver, and urinary bladder at 143.0 ± 80.20, 74.50 ± 34.20, 65.50 ± 29.60, 64.40 ± 22.10, and 27.10 ± 11.70 μSv/MBq, respectively.

**Conclusions:**

The ED for florbetapir has been calculated for nine healthy volunteers. At a dose of 370 MBq florbetapir, the total average ED is approximately 6.88 mSv.

## Background

Accumulation of amyloid-β (Aβ) fibrils in the form of amyloid plaques is a neuropathological requirement for definitive diagnosis [1]. Positron emission tomography (PET) tracers that bind to aggregated Aβ peptides offer promise to directly assess fibrillar amyloid pathology *in vivo*[2,3]. The initial studies with ^11^C Pittsburgh Compound B (PiB) were the first to clearly demonstrate the feasibility of this approach [4]. However, the 20-min half-life of ^11^C limits the use of this molecular imaging ligand to specialized research centers. To address this issue, several amyloid imaging ligands that use the longer-lived ^18^F PET isotope have been developed [5].

One of these tracers is florbetapir F 18 ((*E*)-4(2-(6-(2-(2-(2-[^18^F]fluoroehoxy)ethoxy)ethoxy)pyridine-3-yl)vinyl)-*N*-mathylbenzeamine, also known as ^18^F-AV-45. When applied at tracer concentrations, florbetapir labels Aβ plaques in sections from patients with pathologically confirmed Alzheimer's disease (AD) [6], and PET human studies show increased tracer retention in the brains of those clinically diagnosed with AD [7]. A recently reported autopsy study demonstrated high correspondence of florbetapir PET images to histopathology [8,9]. The present study was carried out to assess the radiation dosimetry of florbetapir.

## Methods

### Subject population

Nine healthy, cognitively normal subjects (three males and six females) with mean age of 58 ± 10 years and mean weight of 81 ± 17 kg were recruited. The study protocol was institutional review board (Biomed IRB, San Diego, CA)-approved, and written informed consent form was obtained from each subject enrolled.

### PET imaging

A catheter was placed in an antecubital vein in the subject's arm for injecting the tracer. Subjects were positioned supine in a Biograph 40 TruePoint PET/CT scanner (Siemens Medical Systems, Malvern, PA, USA) with the arms at their sides. For the purpose of this study, a whole-body (WB) scan was defined as a total of seven bed positions from the subject's top of the head to mid-thighs. Subjects wore an abdominal binder during imaging to reduce respiratory motion between PET and computed tomography (CT) acquisitions. Once positioned, a low-dose whole-body CT scan was obtained and then the subjects were injected with a target dose of 370 MBq florbetapir (actual mean dose 395 ± 27.9 MBq). Following injection, a session of up to four WB PET emission scans was acquired using the initial CT without subject repositioning. Following a short break (<30 min), a second low-dose CT was taken followed immediately by a session of at least two WB PET emission scans. Additional sessions consisting of low-dose CT and PET emission scans were subsequently obtained at roughly 60-min intervals for a total of up to seven sessions with breaks between the sessions for patient comfort. Each session included at least one PET whole-body emission scan (seven bed positions). The time per PET bed position was varied from 90 s for the first session to 4 min for the final session to compensate for radioactive decay.

In general, session 1 consisted of four PET scans, session 2 consisted of two PET scans, and sessions 3, 4, 5, 6, and 7 consisted of one PET scan each. Although scan data was obtained for an average of approximately 6 h (range 4.25 to 7.37 h) for all subjects, to accommodate subject comfort, some scans were omitted in some subjects: For four of nine subjects, all 11 emission scans were acquired; in three subjects, 10 emission scans were acquired; in one subject, 9 emission scans were acquired; and in one subject, the data were acquired in 7 scans. The time required to reposition the bed to initiate subsequent emission scans in a session was variable, and patients were given periodic breaks as requested. This resulted in variable start time across the subjects and variable number of image sequences (Table 1). The mean time for all subjects until the end of the last scan was 5.95 ± 1.09 h.

**Table 1:** CT and PET acquisition time sequence for all nine volunteers: frame start time after injection in minutes

**Session**	**Subject number**								
	**009**	**001**	**012**	**007**	**008**	**010**	**002**	**006**	**003**
Low-dose CT scan after positioning and before florbetapir injection									
1.1	4	9	3	3	4	4	1	3	2
1.2	18	25	16	20	17	22	18	15	20
1.3	29	50	30	33	30	39	NA	26	37
1.4	47	64	45	46	46	55	52	39	NA
Short break <30 min									
Low-dose CT scan after positioning									
2.1	76	105	71	85	72	90	88	64	76
2.2	94	120	88	102	89	109	113	79	102
2.3	NA	NA	NA	NA	NA	NA	133	NA	NA
Low-dose CT scan after positioning									
3.1	137	170	129	129	130	187	206	112	153
Low-dose CT scan after positioning									
4.1	204	236	198	180	186	246	250	181	228
Low-dose CT scan after positioning									
5.1	268	301	241	248	246	306	303	240	NA
Low-dose CT scan after positioning									
6.1	326	363	NA	NA	308	366	364	301	NA
Low-dose CT scan after positioning									
7.1	384	428	NA	360	356	NA	NA	361	NA

NA means that the subject is not imaged during that duration of the session. PET scans are identified as session#.scan#.

#### ***PET data post-processing***

The acquired emission data were reconstructed using iterative reconstruction (two iterations, eight subsets), with attenuation, random, and scatter correction, and a post-reconstruction Gaussian filter of 7 mm was applied. PET whole-body images were generated with a matrix size of 128 × 128 per slice and a voxel size of 5.35 × 5.35 × 3 mm^3^. The camera sensitivity was calibrated using a uniform cylindrical phantom (dimensions: 20-cm diameter, 30-cm length, and 9,420-mL volume) filled with a known concentration of activity to generate a camera-to-well counter cross-calibration factor (approximately 1.1 (Bq/mL)/cps)) which was subsequently applied to all PET measurement of *in vivo* activity.

#### ***Dosimetry analysis***

**Figure 1:**
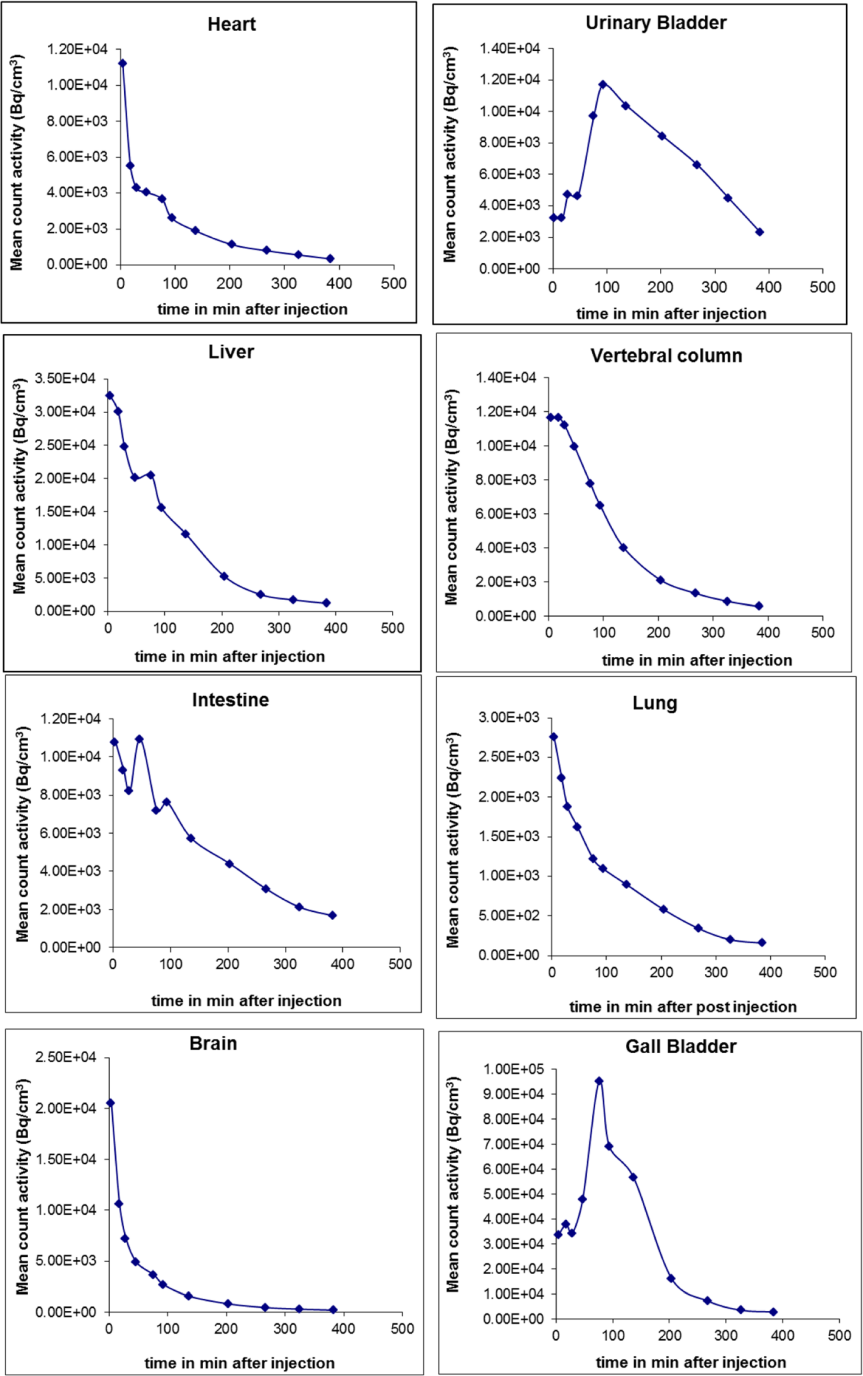
**Time-activity curves showing mean count activity (Bq/cm**^**3**^**) over time in eight organs for a representative subject.** Mean radioactivity concentration values are different across the organs, so the upper limit of the scale is variable.

Following reconstruction, volumes of interest (VOIs) for various body organs were defined on the PET images for each patient. Based on the visual examination, the anatomical information from the CT data and the human whole-body reference atlas [10,11] was used to delineate patient specific VOIs. Organs defined by the VOI process were the brain, liver, lung, heart wall, urinary bladder, vertebrae, intestine, and gallbladder. Using these regions on the PET emission data, average counts for the organs were determined at each time imaged. The regional activities were then calculated for each time point, thus providing a time-activity curve (TAC) for each region of a given subject (Figure 1). All organ TACs were fitted with one or two exponential terms using SAAM II software [12]. Time integrals of activity for all the organs were calculated from a lower limit of zero to an upper limit of infinity and expressed as the number of disintegrations in source organs [13]. These values were entered in OLINDA/EXM software [14], using the adult model. Activity observed in a segment of the gastrointestinal (GI) tract was used to estimate total intestinal excretion, using the ICRP 30 GI kinetic model in the OLINDA/EXM software.

The cumulated activity in the urinary bladder contents was determined by fitting the TAC of the bladder contents with an exponential in-growth function of the form:

(1) $$U\left(t\right)=U\left(0\right)\times \left(1-e^{-t/\tau }\right)$$

where *U*(*t*) is the fraction of activity in the bladder at time ‘*t*’ after injection and *τ* is the half-life of ^18^F/ln [2]. These parameters were entered into the voiding bladder model of OLINDA/EXM. For the principal analysis, no bladder voiding was assumed. A separate analysis was performed with no bladder void until 90 min post-injection.

Additional calculations were performed using the feature in OLINDA/EXM that allows modification of doses on body mass ratios to adjust the calculations to be more appropriate for the subject's individual body size. All organs and the whole body were selected and scaled by ratios of the subject's body weight to that of the reference phantom to develop dose estimates for 50-, 60-, and 80-kg individuals (the reference adult is approximately 70 kg).

## Results

**Figure 2:**
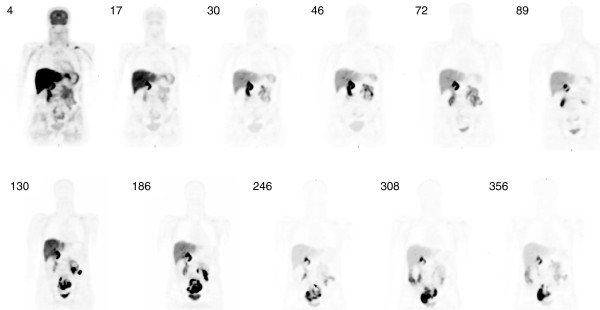
Series of PET whole-body images from 4 to 356 min after injection of florbetapir F 18.

Figure 2 shows a typical series of PET scans in a healthy volunteer enrolled in this study. The series of images demonstrates rapid distribution of florbetapir shortly following injection. Essentially, immediate uptake into, followed by rapid clearance out of, the normal brain was seen. Rapid clearance from circulation and localization in the liver was also observed. Some accumulation was observed in the urinary bladder, but primarily, the elimination appeared to occur by way of clearance from the liver and excretion through the gallbladder into the GI tract.

Cumulated activities in each organ were determined for individual subjects to allow subject-specific dose estimates to be determined. Based on the nine subject measurements of florbetapir, the effective dose (ED) was determined to be 18.60 ± 4.260 μSv/MBq or 6.88 mSv for a 370-MBq dose with no specific assumptions regarding bladder voiding. In a separate analysis assuming no urinary bladder voiding until 90 min, the ED was 18.5 ± 4.23 μSv/MBq or 6.84 mSv for 370 MBq. Tables 2 and 3 summarizes average organ dosimetry for nine subjects with different urinary bladder voiding models. Data for both voiding models shows that the organs that received the highest radiation absorbed dose were the gallbladder, upper large intestine, small intestine, liver, and urinary bladder at 143.0 ± 80.20, 74.50 ± 34.20, 65.50 ± 29.60, 64.40 ± 22.10, and 27.10 ± 11.70 μSv/MBq, respectively.

**Table 2:** Radiation dose estimates (μSv/MBq) with no urinary bladder voiding

**Target organ**	**024-009**	**024-001**	**024-012**	**024-007**	**024-008**	**024-010**	**024-002**	**024-006**	**024-003**	**Average**	**Std Dev**
Adrenals	1.30E + 01	1.24E + 01	1.63E + 01	1.34E + 01	1.42E + 01	1.40E + 01	1.33E + 01	1.38E + 01	1.17E + 01	1.36E + 01	1.29E + 00
Brain	1.27E + 01	7.03E + 00	9.50E + 00	8.25E + 00	1.12E + 01	1.20E + 01	1.10E + 01	9.86E + 00	8.66E + 00	1.00E + 01	1.86E + 00
Breasts	6.76E + 00	6.87E + 00	6.66E + 00	6.98E + 00	4.61E + 00	6.73E + 00	5.09E + 00	5.78E + 00	6.65E + 00	6.24E + 00	8.65E - 01
Gallbladder wall	7.18E + 01	8.30E + 01	2.22E + 02	1.26E + 02	2.00E + 02	8.36E + 01	1.07E + 02	3.02E + 02	8.87E + 01	1.43E + 02	8.02E + 01
LLI wall	3.05E + 01	2.09E + 01	2.19E + 01	3.37E + 01	4.16E + 01	2.90E + 01	4.21E + 01	1.57E + 01	1.51E + 01	2.78E + 01	1.02E + 01
Small intestine	7.08E + 01	4.36E + 01	5.05E + 01	7.91E + 01	1.10E + 02	6.69E + 01	1.07E + 02	3.47E + 01	2.73E + 01	6.55E + 01	2.96E + 01
Stomach wall	1.22E + 01	1.13E + 01	1.22E + 01	1.31E + 01	1.17E + 01	1.22E + 01	1.19E + 01	1.04E + 01	1.03E + 01	1.17E + 01	9.06E - 01
ULI wall	7.96E + 01	4.86E + 01	5.79E + 01	8.93E + 01	1.27E + 02	7.56E + 01	1.22E + 02	4.02E + 01	3.00E + 01	7.45E + 01	3.42E + 01
Heart wall	1.35E + 01	1.06E + 01	1.41E + 01	1.18E + 01	1.55E + 01	1.46E + 01	1.35E + 01	1.27E + 01	1.14E + 01	1.31E + 01	1.59E + 00
Kidneys	1.28E + 01	1.19E + 01	1.46E + 01	1.37E + 01	1.38E + 01	1.33E + 01	1.33E + 01	1.24E + 01	1.09E + 01	1.30E + 01	1.11E + 00
Liver	4.63E + 01	4.18E + 01	9.81E + 01	4.81E + 01	9.25E + 01	6.42E + 01	7.57E + 01	7.41E + 01	3.89E + 01	6.44E + 01	2.21E + 01
Lungs	8.54E + 00	7.69E + 00	9.37E + 00	7.67E + 00	9.34E + 00	9.31E + 00	8.14E + 00	9.19E + 00	7.47E + 00	8.52E + 00	8.01E - 01
Muscle	9.36E + 00	8.87E + 00	8.70E + 00	9.77E + 00	7.60E + 00	9.19E + 00	8.23E + 00	7.56E + 00	8.36E + 00	8.63E + 00	7.62E - 01
Ovaries	1.93E + 01	1.50E + 01	1.50E + 01	2.11E + 01	2.24E + 01	1.86E + 01	2.34E + 01	1.16E + 01	1.23E + 01	1.76E + 01	4.34E + 00
Pancreas	1.37E + 01	1.31E + 01	1.70E + 01	1.47E + 01	1.51E + 01	1.45E + 01	1.40E + 01	1.50E + 01	1.23E + 01	1.44E + 01	1.34E + 00
Red marrow	1.67E + 01	1.26E + 01	1.33E + 01	1.44E + 01	1.66E + 01	1.51E + 01	1.56E + 01	1.28E + 01	1.19E + 01	1.43E + 01	1.78E + 00
Osteogenic cells	3.43E + 01	2.43E + 01	2.43E + 01	2.60E + 01	3.19E + 01	2.94E + 01	2.88E + 01	2.58E + 01	2.38E + 01	2.76E + 01	3.71E + 00
Skin	6.62E + 00	6.51E + 00	5.97E + 00	6.79E + 00	4.47E + 00	6.41E + 00	5.08E + 00	5.23E + 00	6.25E + 00	5.93E + 00	8.09E - 01
Spleen	9.73E + 00	9.53E + 00	9.06E + 00	1.02E + 01	7.28E + 00	9.51E + 00	7.96E + 00	7.86E + 00	8.99E + 00	8.90E + 00	9.86E - 01
Testes	7.75E + 00	7.84E + 00	6.44E + 00	8.20E + 00	4.37E + 00	7.51E + 00	5.93E + 00	5.62E + 00	7.66E + 00	6.81E + 00	1.29E + 00
Thymus	8.11E + 00	8.26E + 00	7.51E + 00	8.30E + 00	4.97E + 00	7.93E + 00	5.73E + 00	6.59E + 00	8.04E + 00	7.27E + 00	1.22E + 00
Thyroid	7.90E + 00	7.98E + 00	6.61E + 00	7.93E + 00	4.09E + 00	7.43E + 00	5.06E + 00	5.96E + 00	7.81E + 00	6.75E + 00	1.43E + 00
Urinary bladder wall	2.25E + 01	1.76E + 01	2.03E + 01	2.89E + 01	2.64E + 01	3.29E + 01	5.47E + 01	1.56E + 01	2.48E + 01	2.71E + 01	1.17E + 01
Uterus	1.68E + 01	1.38E + 01	1.35E + 01	1.85E + 01	1.80E + 01	1.67E + 01	2.03E + 01	1.08E + 01	1.22E + 01	1.56E + 01	3.19E + 00
Total body	1.22E + 01	1.07E + 01	1.22E + 01	1.22E + 01	1.23E + 01	1.22E + 01	1.22E + 01	1.04E + 01	9.96E + 00	1.16E + 01	9.51E - 01
Effective dose	1.90E + 01	1.49E + 01	1.77E + 01	2.01E + 01	2.44E + 01	1.98E + 01	2.49E + 01	1.43E + 01	1.27E + 01	1.86E + 01	4.26E + 00

Std Dev, standard deviation; LLI, lower large intestine; ULI, upper large intestine.

**Table 3:** Radiation dose estimates (μSv/MBq) assuming one urinary bladder void at 90 min post-injection

**Target organ**	**24-009**	**24-001**	**24-012**	**24-007**	**24-008**	**24-010**	**24-002**	**24-006**	**24-003**	**Average**	**Std Dev**
Adrenals	1.30E + 01	1.24E + 01	1.63E + 01	1.34E + 01	1.42E + 01	1.40E + 01	1.33E + 01	1.38E + 01	1.17E + 01	1.36E + 01	1.29E + 00
Brain	1.27E + 01	7.03E + 00	9.50E + 00	8.25E + 00	1.12E + 01	1.20E + 01	1.10E + 01	9.86E + 00	8.66E + 00	1.00E + 01	1.86E + 00
Breasts	6.76E + 00	6.87E + 00	6.66E + 00	6.98E + 00	4.61E + 00	6.73E + 00	5.09E + 00	5.78E + 00	6.65E + 00	6.24E + 00	8.65E - 01
Gallbladder wall	7.18E + 01	8.30E + 01	2.22E + 02	1.26E + 02	2.00E + 02	8.35E + 01	1.07E + 02	3.02E + 02	8.87E + 01	1.43E + 02	8.02E + 01
LLI wall	3.03E + 01	2.09E + 01	2.18E + 01	3.37E + 01	4.15E + 01	2.88E + 01	4.20E + 01	1.57E + 01	1.50E + 01	2.77E + 01	1.02E + 01
Small intestine	7.07E + 01	4.36E + 01	5.04E + 01	7.91E + 01	1.10E + 02	6.69E + 01	1.07E + 02	3.47E + 01	2.73E + 01	6.55E + 01	2.96E + 01
Stomach wall	1.22E + 01	1.13E + 01	1.22E + 01	1.31E + 01	1.17E + 01	1.22E + 01	1.19E + 01	1.03E + 01	1.03E + 01	1.17E + 01	9.24E - 01
ULI wall	7.96E + 01	4.86E + 01	5.79E + 01	8.93E + 01	1.26E + 02	7.55E + 01	1.22E + 02	4.02E + 01	3.00E + 01	7.43E + 01	3.40E + 01
Heart wall	1.35E + 01	1.06E + 01	1.41E + 01	1.18E + 01	1.55E + 01	1.46E + 01	1.35E + 01	1.27E + 01	1.14E + 01	1.31E + 01	1.59E + 00
Kidneys	1.28E + 01	1.19E + 01	1.46E + 01	1.37E + 01	1.38E + 01	1.33E + 01	1.33E + 01	1.24E + 01	1.09E + 01	1.30E + 01	1.11E + 00
Liver	4.63E + 01	4.18E + 01	9.81E + 01	4.81E + 01	9.25E + 01	6.42E + 01	7.57E + 01	7.41E + 01	3.89E + 01	6.44E + 01	2.21E + 01
Lungs	8.54E + 00	7.69E + 00	9.37E + 00	7.67E + 00	9.34E + 00	9.31E + 00	8.14E + 00	9.18E + 00	7.47E + 00	8.52E + 00	7.99E - 01
Muscle	9.31E + 00	8.87E + 00	8.68E + 00	9.78E + 00	7.57E + 00	9.15E + 00	8.22E + 00	7.55E + 00	8.33E + 00	8.61E + 00	7.63E - 01
Ovaries	1.91E + 01	1.50E + 01	1.49E + 01	2.11E + 01	2.23E + 01	1.84E + 01	2.34E + 01	1.16E + 01	1.22E + 01	1.76E + 01	4.33E + 00
Pancreas	1.37E + 01	1.31E + 01	1.70E + 01	1.47E + 01	1.50E + 01	1.45E + 01	1.40E + 01	1.50E + 01	1.23E + 01	1.44E + 01	1.34E + 00
Red marrow	1.67E + 01	1.26E + 01	1.33E + 01	1.44E + 01	1.66E + 01	1.51E + 01	1.56E + 01	1.28E + 01	1.19E + 01	1.43E + 01	1.78E + 00
Osteogenic cells	3.43E + 01	2.43E + 01	2.43E + 01	2.60E + 01	3.19E + 01	2.94E + 01	2.88E + 01	2.58E + 01	2.38E + 01	2.76E + 01	3.71E + 00
Skin	6.60E + 00	6.51E + 00	5.96E + 00	6.79E + 00	4.46E + 00	6.39E + 00	5.08E + 00	5.23E + 00	6.24E + 00	5.92E + 00	8.07E - 01
Spleen	9.72E + 00	9.53E + 00	9.06E + 00	1.03E + 01	7.27E + 00	9.50E + 00	7.96E + 00	7.86E + 00	8.98E + 00	8.91E + 00	1.00E + 00
Testes	7.62E + 00	7.84E + 00	6.37E + 00	8.22E + 00	4.29E + 00	7.39E + 00	5.89E + 00	5.58E + 00	7.57E + 00	6.75E + 00	1.30E + 00
Thymus	8.11E + 00	8.26E + 00	7.51E + 00	8.30E + 00	4.97E + 00	7.93E + 00	5.73E + 00	6.59E + 00	8.04E + 00	7.27E + 00	1.22E + 00
Thyroid	7.90E + 00	7.98E + 00	6.61E + 00	7.93E + 00	4.09E + 00	7.43E + 00	5.06E + 00	5.96E + 00	7.81E + 00	6.75E + 00	1.43E + 00
Urinary bladder wall	1.54E + 01	1.76E + 01	1.65E + 01	2.98E + 01	2.21E + 01	2.67E + 01	5.28E + 01	1.38E + 01	2.01E + 01	2.39E + 01	1.21E + 01
Uterus	1.64E + 01	1.38E + 01	1.33E + 01	1.86E + 01	1.78E + 01	1.63E + 01	2.02E + 01	1.06E + 01	1.19E + 01	1.54E + 01	3.22E + 00
Total body	1.21E + 01	1.07E + 01	1.22E + 01	1.22E + 01	1.22E + 01	1.22E + 01	1.22E + 01	1.04E + 01	9.93E + 00	1.16E + 01	9.41E - 01
Effective dose	1.86E + 01	1.49E + 01	1.75E + 01	2.01E + 01	2.41E + 01	1.94E + 01	2.48E + 01	1.42E + 01	1.25E + 01	1.85E + 01	4.23E + 00
Adjusted bladder AUC	1.10E + 01	1.70E + 01	1.70E + 01	4.00E + 01	3.00E + 01	3.60E + 01	9.30E + 01	1.40E + 01	2.40E + 01	3.13E + 01	2.52E + 01

The organ volume, organ mass, and whole body mass were selected and scaled [15] by ratios to the 70-kg reference phantom model to obtain dose estimates for 50-, 60-, and 80-kg individuals. Average dose estimates for different body weights are summarized in Table 4.

**Table 4:** Average dose estimates (μSv/MBq) across nine healthy volunteers for 50-, 60-, and 80-kg scaled weights

**Target organ**	**50 kg**		**60 kg**		**70 kg**		**80 kg**	
	**Average**	**Std Dev**	**Average**	**Std Dev**	**Average**	**Std Dev**	**Average**	**Std Dev**
Adrenals	1.75E + 01	1.65E + 00	1.52E + 01	1.38E + 00	1.36E + 01	1.30E + 00	1.23E + 01	1.22E + 00
Brain	1.33E + 01	2.51E + 00	1.14E + 01	2.13E + 00	1.00E + 01	1.86E + 00	9.01E + 00	1.67E + 00
Breasts	8.17E + 00	1.22E + 00	7.05E + 00	1.00E + 00	6.23E + 00	8.62E - 01	5.63E + 00	7.68E - 01
Gallbladder wall	1.65E + 02	8.76E + 01	1.48E + 02	8.19E + 01	1.43E + 02	8.02E + 01	1.39E + 02	7.86E + 01
LLI wall	3.19E + 01	1.09E + 01	2.96E + 01	1.05E + 01	2.78E + 01	1.02E + 01	2.65E + 01	9.91E + 00
Small intestine	7.10E + 01	3.09E + 01	6.81E + 01	3.03E + 01	6.55E + 01	2.96E + 01	6.36E + 01	2.92E + 01
Stomach wall	2.24E + 01	2.31E + 01	1.31E + 01	9.96E - 01	1.17E + 01	8.99E - 01	1.06E + 01	8.34E - 01
ULI wall	7.52E + 01	3.99E + 01	7.80E + 01	3.52E + 01	7.44E + 01	3.42E + 01	7.17E + 01	3.33E + 01
Heart wall	1.72E + 01	2.06E + 00	1.48E + 01	1.81E + 00	1.27E + 01	1.86E + 00	1.15E + 01	1.67E + 00
Kidneys	1.67E + 01	1.45E + 00	1.45E + 01	1.17E + 00	1.30E + 01	1.11E + 00	1.18E + 01	1.02E + 00
Liver	8.59E + 01	2.94E + 01	7.40E + 01	2.48E + 01	6.44E + 01	2.21E + 01	5.77E + 01	1.98E + 01
Lungs	1.10E + 01	9.93E - 01	9.58E + 00	8.91E - 01	8.52E + 00	8.01E - 01	7.73E + 00	7.21E - 01
Muscle	1.11E + 01	1.06E + 00	9.70E + 00	8.76E - 01	8.62E + 00	7.59E - 01	7.82E + 00	6.79E - 01
Ovaries	2.20E + 01	5.15E + 00	1.97E + 01	4.79E + 00	1.76E + 01	4.32E + 00	1.61E + 01	3.96E + 00
Pancreas	1.90E + 01	2.91E + 00	1.61E + 01	1.42E + 00	1.44E + 01	1.35E + 00	1.31E + 01	1.24E + 00
Red marrow	1.96E + 01	2.24E + 00	1.65E + 01	1.96E + 00	1.43E + 01	1.76E + 00	1.27E + 01	1.63E + 00
Osteogenic cells	4.01E + 01	4.90E + 00	3.27E + 01	4.21E + 00	2.76E + 01	3.71E + 00	2.40E + 01	3.34E + 00
Skin	7.75E + 00	1.11E + 00	6.70E + 00	9.32E - 01	5.92E + 00	8.06E - 01	5.34E + 00	7.15E - 01
Spleen	1.19E + 01	2.21E + 00	1.00E + 01	1.14E + 00	8.90E + 00	9.80E - 01	8.07E + 00	8.85E - 01
Testes	8.84E + 00	1.70E + 00	7.68E + 00	1.47E + 00	6.81E + 00	1.29E + 00	6.16E + 00	1.16E + 00
Thymus	9.44E + 00	1.64E + 00	8.19E + 00	1.39E + 00	7.26E + 00	1.21E + 00	6.57E + 00	1.09E + 00
Thyroid	8.79E + 00	1.90E + 00	7.61E + 00	1.62E + 00	6.75E + 00	1.43E + 00	6.10E + 00	1.28E + 00
Urinary bladder wall	3.10E + 01	1.25E + 01	2.88E + 01	1.21E + 01	2.71E + 01	1.17E + 01	2.58E + 01	1.14E + 01
Uterus	1.97E + 01	3.84E + 00	1.74E + 01	3.55E + 00	1.56E + 01	3.19E + 00	1.42E + 01	2.96E + 00
Total body	1.50E + 01	1.14E + 00	1.30E + 01	1.02E + 00	1.16E + 01	9.43E - 01	1.05E + 01	8.76E - 01
Effective dose	2.37E + 01	5.50E + 00	2.06E + 01	4.55E + 00	1.86E + 01	4.26E + 00	1.72E + 01	4.04E + 00

Calculated by scaling organs and whole body from the 70-kg adult reference model.

## Discussion

The present study was designed to provide the bio-distribution and radiation dosimetry of florbetapir. Our study has resulted in an ED estimate of 18.6 ± 4.26 μSv/MBq or 6.88 mSv for 370 MBq for PET. We found that florbetapir is rapidly distributed throughout the body shortly following I.V. administration. The gallbladder was the organ that received the highest absorbed dose, with an average value of 143.0 ± 80.20 μSv/MBq across nine healthy volunteers. Variability in gallbladder activity was observed, which is possibly related to differences in the individual kinetics of gallbladder emptying or differences across subjects in the timing of food consumption or diet. Images over time show that elimination occurs primarily by way of clearance from the liver and excretion through the gallbladder into the GI tract. Some accumulation is also observed in the urinary bladder. Modeling urinary bladder voiding at 90 min post-injection did not substantially change the radiation dosimetry results.

The present findings are very similar to the results of a preliminary study [16] that calculated an effective dose of 19.3 ±1.30 μSv/MBq in three subjects from an Asian population and evaluated the organ kinetics over a shorter (3 h) time period. In the present study, the radiation dosimetry of florbetapir was studied in nine healthy volunteers from an American population and the organ kinetics were evaluated for approximately 6 h. Radiation dosimetry of other ^18^F-labeled PET amyloid tracers has been evaluated by a methodology similar to that of the present study. Koole et al. [17] and O'Keefe et al. [18] previously studied ^18^F-GE067 (flutemetamol) and ^18^F-BAY94-9172 (florbetaben) radiation dosimetries, respectively. O'Keefe et al. [18] also reported the radiation dose estimate for the ^11^C-labeled PET amyloid tracer ^11^C-PIB. The International Commission on Radiological Protection (ICRP), in its Publication 106 [15], presents tables of ^18^F-fluorodeoxyglucose (^18^F-FDG) and reports the effective dose. The ICRP authors derived a kinetic model based on the data published by Hays and Segall [19], Deloar et al. [20], and Meija et al. [21]. The retention in the specified source organs was considered to be infinite. The ED calculated for florbetapir, flutemetamol [^18^F-GE067], florbetaben [^18^F-BAY94-9172], ^18^F-FDG, and ^11^C-PIB are summarized in Table 5.

**Table 5:** **Comparison of ED (adult phantom model) estimates for florbetapir F 18 and other **^**18**^**F-labeled pharmaceuticals**

**Tracer**	**ED (μSv/MBq)**
Florbetapir F 18	18.60
Flutemetamol [^18^F-GE067] [17]	33.8
Florbetaben [^18^F-BAY94-9172] [18]	14.7
^18^F-FDG [15]	19.0
^11^C-PIB [18]	5.29

## Conclusions

The radiation dosimetry for the ^18^F-labeled amyloid imaging agent florbetapir has been calculated in nine healthy volunteers and results in an effective radiation dose of 18.60 ± 4.26 μSv/MBq or 6.88 mSv for 370 MBq.

## Competing interests

Abhinay D. Joshi, Michael J. Pontecorvo, Alan P. Carpenter, Mark A. Mintun, and Daniel M. Skovronsky are employees of Avid Radiopharmaceuticals, Inc. Lee P. Adler has no relevant financial disclosures other than the clinical study contract with Avid for this work. Michael G. Stabin has no relevant financial disclosures other than a consultant with Avid for this work.

## Authors’ contributions

ADJ participated in the study design, image analysis, and data interpretation and drafted the manuscript. MJP was responsible for the study concept and design, data interpretation and review of the manuscript. LA carried out the data acquisition and participated in the scientific review. MGS performed the dosimetry analysis. APC was responsible for the administrative and material support, data interpretation and critical review of the manuscript. DMS supervised the study, provided administrative or material support and participated in the critical review. MAM participated in the data interpretation and critical review of the manuscript. All authors read and approved the final manuscript.
